# Firearm Homicides and Suicides in Major Metropolitan Areas — United States, 2012–2013 and 2015–2016

**DOI:** 10.15585/mmwr.mm6744a3

**Published:** 2018-11-09

**Authors:** Scott R. Kegler, Linda L. Dahlberg, James A. Mercy

**Affiliations:** ^1^Division of Analysis, Research, and Practice Integration, National Center for Injury Prevention and Control, CDC; ^2^Division of Violence Prevention, National Center for Injury Prevention and Control, CDC.

Firearm homicides and suicides represent a continuing public health concern in the United States. During 2015–2016, a total of 27,394 firearm homicides (including 3,224 [12%] among persons aged 10–19 years) and 44,955 firearm suicides (including 2,118 [5%] among persons aged 10–19 years) occurred among U.S. residents (*1*). This report updates an earlier report (*2*) that provided statistics on firearm homicides and suicides in major metropolitan areas during 2006–2007 and 2009–2010, and places continued emphasis on youths, in recognition of the importance of early prevention efforts. Firearm homicide and suicide rates were determined for the 50 most populous U.S. metropolitan statistical areas (MSAs)* during 2012–2013 and 2015–2016 using mortality data from the National Vital Statistics System (NVSS) and population data from the U.S. Census Bureau. In contrast to the earlier report, which indicated that firearm homicide rates among persons of all ages had been declining both nationally and in large MSAs overall, current findings show that rates have returned to levels comparable to those observed during 2006–2007. Consistent with the earlier report, these findings show that firearm suicide rates among persons aged ≥10 years have continued to increase, both nationally and in large MSAs overall. Although firearm suicide rates among youths remain notably lower than those among persons of all ages, youth rates have also increased both nationally and in large MSAs collectively. These findings can inform ongoing development and monitoring of strategies directed at reducing firearm-related violence.

NVSS mortality data for 2012–2013 and 2015–2016 were used to identify firearm homicides (*International Classification of Diseases, 10th Revision* [ICD-10] underlying cause codes X93–X95 and U01.4 [U.S. extension to ICD-10]) and firearm suicides (codes X72–X74) among U.S. residents. Firearm homicide and suicide counts were tabulated for county groupings forming the 50 largest MSAs (by population rank mid-year 2016).^†^ Tabulated counts were integrated with U.S. Census Bureau population estimates for the counties forming these MSAs to calculate annual firearm homicide rates for persons of all ages and annual firearm suicide rates for persons aged ≥10 years (persons aged <10 years were excluded because intent for self-harm often is not attributed to young children). Rates were similarly calculated for youths aged 10–19 years. Rates among persons of all ages were age-adjusted to the year 2000 U.S. standard population. MSA-level data involving firearm homicide or suicide counts <20 are not reported separately because of concerns related to statistical reliability (stability) and data privacy. However, such data were included in the calculations for all large MSAs combined.

The rates of firearm homicide among persons of all ages during 2015–2016 varied widely among the 50 largest MSAs, ranging from 1.1 (Providence-Warwick) to 16.6 (New Orleans-Metairie) per 100,000 residents per year (Table). The rate for all large MSAs combined was 4.9, compared with a national rate of 4.4. This represents an increase from 2012–2013, when the rate for large MSAs combined was 4.1 and the national rate was 3.7. Between 2012–2013 and 2015–2016, firearm homicide rates increased for 43 (86%) of the 50 large MSAs considered individually. Among youths, the firearm homicide rate for large MSAs combined was 4.7 during 2015–2016, compared with a national rate of 3.9. Similar to rates among persons of all ages, this represents an increase from 2012–2013, when the rate for large MSAs combined was 4.3 and the national rate was 3.4. Males accounted for approximately 85% of firearm homicide victims (all ages) during both reporting periods, for the 50 largest MSAs combined as well as nationally.

**TABLE:** Numbers and annual rates (per 100,000 population) of firearm homicides and suicides for the 50 most populous metropolitan statistical areas (MSAs) — United States, 2012–2013 and 2015–2016*

MSA (ordered alphabetically)	Years	Firearm homicides		Firearm suicides	
		All ages	Aged 10–19 years	Aged ≥10 years	Aged 10–19 years
		No.^†^ (rate^§^)	No. (rate)	No.^†^ (rate^§^)	No. (rate)
**U.S. total**	**2012–2013**	**22,822 (3.7)**	**2,858 (3.4)**	**41,833 (7.4)**	**1,736 (2.1)**
	**2015–2016**	**27,392 (4.4)**	**3,224 (3.9)**	**44,950 (7.7)**	**2,118 (2.5)**
MSA total (50 MSAs)^¶^	2012–2013	14,086 (4.1)	1,951 (4.3)	17,339 (5.6)	671 (1.5)
	2015–2016	17,128 (4.9)	2,153 (4.7)	18,513 (5.8)	851 (1.9)
Atlanta, Sandy Springs, Roswell (Georgia)	2012–2013	536 (4.9)	69 (4.4)	726 (7.7)	35 (2.2)
	2015–2016	717 (6.3)	106 (6.5)	764 (7.6)	48 (2.9)
Austin, Round Rock (Texas)	2012–2013	39 (1.0)	—** (—)	254 (8.2)	— (—)
	2015–2016	99 (2.3)	— (—)	283 (8.2)	— (—)
Baltimore, Columbia, Towson (Maryland)	2012–2013	422 (7.7)	46 (6.5)	262 (5.2)	— (—)
	2015–2016	656 (12.2)	63 (9.1)	239 (4.6)	— (—)
Birmingham, Hoover (Alabama)	2012–2013	187 (8.6)	23 (7.8)	230 (11.2)	— (—)
	2015–2016	275 (12.6)	23 (7.9)	245 (11.9)	— (—)
Boston, Cambridge, Newton (Massachusetts, New Hampshire)	2012–2013	112 (1.1)	— (—)	177 (2.1)	— (—)
	2015–2016	113 (1.2)	— (—)	179 (2.1)	— (—)
Buffalo, Cheektowaga, Niagara Falls (New York)	2012–2013	86 (4.0)	— (—)	85 (4.0)	— (—)
	2015–2016	81 (3.6)	— (—)	76 (3.3)	— (—)
Charlotte, Concord, Gastonia (North Carolina, South Carolina)	2012–2013	197 (4.3)	26 (4.1)	285 (6.9)	— (—)
	2015–2016	231 (4.8)	23 (3.4)	348 (8.1)	22 (3.3)
Chicago, Naperville, Elgin (Illinois, Indiana, Wisconsin)	2012–2013	1,137 (6.0)	244 (9.3)	610 (3.6)	27 (1.0)
	2015–2016	1,527 (8.1)	272 (10.7)	620 (3.6)	29 (1.1)
Cincinnati (Ohio, Kentucky, Indiana)	2012–2013	160 (4.0)	29 (4.9)	264 (6.9)	— (—)
	2015–2016	174 (4.2)	31 (5.3)	307 (8.1)	22 (3.8)
Cleveland, Elyria (Ohio)	2012–2013	193 (5.1)	38 (7.0)	256 (6.8)	— (—)
	2015–2016	298 (7.8)	33 (6.4)	277 (7.2)	— (—)
Columbus (Ohio)	2012–2013	180 (4.5)	22 (4.2)	259 (7.6)	— (—)
	2015–2016	206 (5.0)	33 (6.2)	256 (7.0)	— (—)
Dallas, Fort Worth, Arlington (Texas)	2012–2013	486 (3.5)	49 (2.5)	857 (7.6)	40 (2.0)
	2015–2016	541 (3.8)	62 (3.0)	949 (7.9)	54 (2.6)
Denver, Aurora, Lakewood (Colorado)	2012–2013	125 (2.3)	— (—)	411 (8.7)	— (—)
	2015–2016	173 (3.0)	— (—)	469 (9.6)	24 (3.3)
Detroit, Warren, Dearborn (Michigan)	2012–2013	764 (9.5)	93 (8.0)	511 (6.6)	28 (2.4)
	2015–2016	652 (8.2)	50 (4.5)	554 (7.0)	28 (2.5)
Hartford, West Hartford, East Hartford (Connecticut)	2012–2013	52 (2.2)	— (—)	68 (3.1)	— (—)
	2015–2016	55 (2.5)	— (—)	59 (2.5)	— (—)
Houston, The Woodlands, Sugar Land (Texas)	2012–2013	626 (4.9)	76 (4.2)	739 (7.2)	43 (2.4)
	2015–2016	828 (6.1)	109 (5.7)	921 (8.2)	45 (2.3)
Indianapolis, Carmel, Anderson (Indiana)	2012–2013	235 (6.2)	27 (5.0)	295 (8.8)	— (—)
	2015–2016	298 (7.7)	45 (8.3)	308 (8.9)	— (—)
Jacksonville (Florida)	2012–2013	186 (6.8)	— (—)	282 (11.6)	— (—)
	2015–2016	208 (7.4)	32 (8.8)	299 (11.0)	— (—)
Kansas City (Missouri, Kansas)	2012–2013	262 (6.6)	27 (4.9)	348 (9.7)	— (—)
	2015–2016	327 (8.2)	38 (6.8)	375 (10.4)	22 (4.0)
Las Vegas, Henderson, Paradise (Nevada)	2012–2013	124 (3.1)	— (—)	371 (10.4)	— (—)
	2015–2016	234 (5.6)	26 (4.8)	391 (10.3)	— (—)
Los Angeles, Long Beach, Anaheim (California)	2012–2013	916 (3.4)	141 (4.0)	769 (3.4)	— (—)
	2015–2016	1,003 (3.7)	123 (3.6)	781 (3.2)	25 (0.7)
Louisville/Jefferson County (Kentucky, Indiana)	2012–2013	113 (4.7)	— (—)	230 (10.3)	— (—)
	2015–2016	204 (8.4)	25 (7.8)	259 (11.0)	— (—)
Memphis (Tennessee, Mississippi, Arkansas)	2012–2013	300 (11.2)	48 (12.3)	188 (8.0)	— (—)
	2015–2016	398 (15.0)	52 (14.0)	183 (7.9)	— (—)
Miami, Fort Lauderdale, West Palm Beach (Florida)	2012–2013	641 (5.7)	94 (6.8)	613 (5.4)	— (—)
	2015–2016	669 (5.9)	98 (7.1)	613 (5.3)	— (—)
Milwaukee, Waukesha, West Allis (Wisconsin)	2012–2013	182 (6.0)	29 (6.8)	176 (6.2)	— (—)
	2015–2016	267 (8.9)	30 (7.2)	182 (6.5)	— (—)
Minneapolis, St. Paul, Bloomington (Minnesota, Wisconsin)	2012–2013	117 (1.7)	— (—)	354 (5.8)	20 (2.2)
	2015–2016	136 (2.0)	26 (2.8)	316 (5.1)	20 (2.2)
Nashville-Davidson, Murfreesboro, Franklin (Tennessee)	2012–2013	129 (3.6)	— (—)	298 (9.6)	— (—)
	2015–2016	178 (4.8)	29 (6.1)	334 (10.2)	23 (4.8)
New Orleans, Metairie (Louisiana)	2012–2013	442 (18.2)	75 (24.4)	151 (7.0)	— (—)
	2015–2016	404 (16.6)	54 (17.6)	186 (8.1)	— (—)
New York, Newark, Jersey City (New York, New Jersey, Pennsylvania)	2012–2013	920 (2.3)	105 (2.1)	602 (1.6)	21 (0.4)
	2015–2016	937 (2.4)	97 (2.0)	564 (1.5)	— (—)
Oklahoma City (Oklahoma)	2012–2013	150 (5.7)	— (—)	253 (11.1)	— (—)
	2015–2016	163 (6.0)	21 (5.7)	317 (13.5)	20 (5.5)
Orlando, Kissimmee, Sanford (Florida)	2012–2013	171 (3.7)	20 (3.4)	262 (6.5)	— (—)
	2015–2016	251 (5.1)	23 (3.7)	275 (6.2)	— (—)
Philadelphia, Camden, Wilmington (Pennsylvania, New Jersey, Delaware, Maryland)	2012–2013	762 (6.5)	107 (6.7)	497 (4.5)	— (—)
	2015–2016	800 (6.8)	94 (6.1)	513 (4.5)	— (—)
Phoenix, Mesa, Scottsdale (Arizona)	2012–2013	344 (4.0)	36 (3.0)	756 (10.0)	28 (2.3)
	2015–2016	397 (4.4)	42 (3.3)	865 (10.6)	34 (2.7)
Pittsburgh (Pennsylvania)	2012–2013	201 (4.7)	39 (7.0)	341 (7.4)	— (—)
	2015–2016	233 (5.4)	38 (7.2)	381 (8.7)	— (—)
Portland, Vancouver, Hillsboro (Oregon, Washington)	2012–2013	60 (1.3)	— (—)	355 (8.5)	— (—)
	2015–2016	80 (1.7)	— (—)	356 (8.2)	— (—)
Providence, Warwick (Rhode Island, Massachusetts)	2012–2013	41 (1.3)	— (—)	79 (2.8)	— (—)
	2015–2016	38 (1.1)	— (—)	103 (3.3)	— (—)
Raleigh (North Carolina)	2012–2013	51 (2.1)	— (—)	117 (6.0)	— (—)
	2015–2016	64 (2.5)	— (—)	121 (5.4)	— (—)
Richmond (Virginia)	2012–2013	129 (5.2)	— (—)	202 (9.2)	— (—)
	2015–2016	180 (7.3)	— (—)	218 (9.2)	— (—)
Riverside, San Bernardino, Ontario (California)	2012–2013	292 (3.3)	32 (2.3)	400 (5.5)	— (—)
	2015–2016	303 (3.3)	41 (3.0)	408 (5.4)	20 (1.5)
Sacramento, Roseville, Arden-Arcade (California)	2012–2013	135 (3.1)	24 (4.0)	252 (6.4)	— (—)
	2015–2016	162 (3.7)	21 (3.5)	259 (6.1)	— (—)
St. Louis (Missouri, Illinois)	2012–2013	355 (6.7)	58 (7.9)	413 (8.2)	20 (2.7)
	2015–2016	596 (11.4)	61 (8.6)	442 (8.7)	— (—)
Salt Lake City (Utah)	2012–2013	29 (1.3)	— (—)	216 (11.8)	— (—)
	2015–2016	46 (1.9)	— (—)	237 (12.4)	20 (5.7)
San Antonio, New Braunfels (Texas)	2012–2013	175 (3.9)	— (—)	299 (7.6)	— (—)
	2015–2016	266 (5.6)	27 (3.9)	305 (7.3)	20 (2.9)
San Diego, Carlsbad (California)	2012–2013	96 (1.4)	— (—)	315 (5.6)	— (—)
	2015–2016	103 (1.6)	— (—)	282 (4.8)	— (—)
San Francisco, Oakland, Hayward (California)	2012–2013	386 (4.5)	72 (7.1)	260 (3.1)	— (—)
	2015–2016	414 (4.5)	60 (5.8)	263 (3.0)	— (—)
San Jose, Sunnyvale, Santa Clara (California)	2012–2013	71 (1.9)	21 (4.4)	85 (2.5)	— (—)
	2015–2016	58 (1.5)	— (—)	97 (2.7)	— (—)
Seattle, Tacoma, Bellevue (Washington)	2012–2013	125 (1.7)	— (—)	425 (6.6)	— (—)
	2015–2016	165 (2.2)	32 (3.7)	452 (6.7)	29 (3.3)
Tampa, St. Petersburg, Clearwater (Florida)	2012–2013	170 (3.1)	— (—)	478 (8.7)	— (—)
	2015–2016	204 (3.7)	21 (3.1)	568 (9.5)	— (—)
Virginia Beach, Norfolk, Newport News (Virginia, North Carolina)	2012–2013	174 (4.8)	20 (4.5)	223 (7.4)	— (—)
	2015–2016	247 (6.8)	38 (8.8)	263 (8.3)	— (—)
Washington, DC, Arlington, Alexandria (District of Columbia, Virginia, Maryland, West Virginia)	2012–2013	300 (2.5)	48 (3.2)	440 (4.3)	— (—)
	2015–2016	469 (3.8)	42 (2.7)	451 (4.2)	28 (1.8)

* Numbers and rates reflect victim place of residence, not place of occurrence.

^†^ These national and MSA-specific numbers exclude a small fraction of records with undocumented decedent age (10 firearm homicides; 11 firearm suicides) and might therefore differ slightly from numbers in the text.

^§^ All-ages rates are age-adjusted to the year 2000 United States standard population.

^¶^ This table includes only the 50 most populous MSAs among the 383 U.S. MSAs currently delineated and therefore cannot be used to establish comprehensive national rankings.

** Dash indicates suppressed entry because of statistical instability or data confidentiality concerns (both associated with small numbers).

Firearm suicide rates among persons of all ages during 2015–2016 also varied widely by large MSA, ranging from 1.5 (New York-Newark-Jersey City) to 13.5 (Oklahoma City) per 100,000 residents per year (Table). The rate for large MSAs combined was 5.8, compared with a national rate of 7.7, representing an increase from 2012–2013, when the rate for large MSAs combined was 5.6 and the national rate was 7.4. Firearm suicide rates among youths remained much lower than those among all persons aged ≥10 years. The rate for this age group for large MSAs combined was 1.9 during 2015–2016, compared with a national rate of 2.5. This also represents an increase from 2012–2013, when the rate for large MSAs combined was 1.5 and the national rate was 2.1. Similar to firearm homicides, males accounted for approximately 85% of firearm suicides (all ages) in both reporting periods, for the 50 largest MSAs combined and nationally.

## Discussion

During 2015–2016, homicide was the 16th leading cause of death among persons of all ages in the United States and the third leading cause among youths aged 10–19 years; a firearm injury was the underlying cause of death in 74% of all homicides and in 87% of youth homicides (*1*). Previously observed decreases in firearm homicide rates have not continued, with more recent rates showing an increase both nationally and in large MSAs considered collectively. Firearm homicide rates among persons of all ages and among youths in the large MSAs overall have both remained higher than corresponding national rates.

During the same period, suicide was the 10th leading cause of death nationally among all persons aged ≥10 years and the second leading cause among youths; a firearm injury was the underlying cause of death in 50% of all suicides and in 42% of youth suicides (*1*). Previously observed increases in firearm suicide rates among persons of all ages continued in recent years, both nationally and in large MSAs collectively; youth firearm suicide rates also increased both nationally and in large MSAs overall. In contrast to firearm homicide rates, firearm suicide rates among persons of all ages and among youths in the large MSAs overall have both remained lower than corresponding national rates. This is consistent with previous research showing that rates of suicide, considering all causes, have been persistently lower in more urban areas than in less urban areas (*3*).

It is too soon to know whether recent increases in firearm homicide rates represent a short-term fluctuation or the beginning of a longer-term trend. From 2015 to 2016, violent crime increased 3.8% for the nation overall, 6.1% in cities with populations ≥250,000, 2.2% in suburban areas and 1.6% in nonmetropolitan counties,^§^ suggesting a short-term increase concentrated particularly in the core cities of metropolitan areas. Preventing firearm homicides can be a challenge for cities across the country; however, previous research has demonstrated that efforts to modify the physical and social environments in cities through abandoned building and vacant lot remediation, greening activities, street outreach and community norm change, low-income housing tax credits, and business improvement districts are significantly associated with reductions in gun assaults, youth homicide, and other violent crime (*4*).

In contrast to homicide rates, which began increasing only recently, rates of suicide in the United States have been gradually increasing over the past decade and a half, across states, population groups, and in rural and urban settings (*3*,*5*,*6*). Rates of firearm suicide, in particular, began increasing coincident with the economic downturn of 2007–2008 and have continued to increase, despite subsequent economic recovery. After declining 7% from 1999 to 2006, annual rates of firearm suicide increased 21% from 2006 to 2016 (from 6.5 to 7.8 per 100,000 residents aged ≥10 years) (*1*). Urban areas recovered more quickly from the economic downturn than did rural areas, but the continued increase in rates of firearm suicide in large MSAs suggests that multiple factors are involved, and that a combination of prevention approaches might be necessary to reduce risks. Efforts to strengthen household financial security; stabilize housing; teach youths coping and problem-solving skills; identify and support persons at risk; and implement proactive prevention policies in schools, workplaces, and other organizational settings are associated with reductions in suicide, suicide attempts, and/or co-occurring risks such as substance abuse, depression, and social isolation (*7*).

Another factor likely affecting both firearm homicide and suicide is access to firearms by persons at risk for harming themselves or others. Previous studies have shown that the interval between deciding to act and attempting suicide can be as brief as 10 minutes or less, and that persons tend not to substitute a different method when a highly lethal method is unavailable or difficult to access (*8*,*9*). Reducing access to lethal means during an acute suicidal crisis by safely storing firearms or temporarily removing them from the home can help reduce suicide risk, particularly among youths (*7*). Preventing persons convicted of or under a restraining order for domestic violence from possessing a firearm has been associated with reductions in intimate partner-related homicide, including firearm homicide (*10*). Efforts to strengthen the background check system to better identify persons convicted of violent crimes or at risk for harming themselves or others might also prevent lethal firearm violence, although these policies need further study (*10*).

The findings in this report are subject to at least two limitations. First, although statistics on nonfatal injuries associated with firearm assault or self-harm might have strengthened the report, population-based nonfatal injury data are not available for MSAs. Second, and notwithstanding the intended focus on youth firearm violence, a more expansive analysis might have addressed firearm homicide and suicide rates for other age groups not separately considered in this report.

Understanding the patterns, characteristics, and impact of firearm violence is an important factor in preventing injuries and deaths. Ongoing tracking of firearm homicide and suicide rates at all geographic levels can provide important input for initiatives directed at reducing firearm-related violence.

**Summary**

**What is already known about this topic?**

Firearm homicide rates in large metro areas are generally higher than for the nation overall, but rates for both had been declining. In contrast, firearm suicide rates in large metro areas are generally lower than those for the nation overall, but rates for both had been increasing.

**What is added by this report?**

Recently, firearm homicide rates in large metro areas and the nation overall began increasing, reaching levels comparable to those a decade ago. Firearm suicide rates have continued to increase in large metro areas and the nation overall.

**What are the implications for public health practice?**

Ongoing tracking of rates at all geographic levels can help support initiatives directed at reducing firearm-related violence.

## Footnotes

* An MSA is defined by the U.S. Office of Management and Budget (OMB) as consisting of “at least one urbanized area of 50,000 or more population, plus adjacent territory that has a high degree of social and economic integration with the core as measured by commuting ties.” This report is based on the revised geographic delineations for MSAs issued by OMB in August 2017.

† The same MSAs were the 50 most populous during both reporting periods; rankings by total population changed slightly. This group of MSAs includes most metropolitan areas with a resident population of at least one million, and currently represents approximately 55% of the U.S. resident population.

§ U.S. Department of Justice, Federal Bureau of Investigation. Crime in the United States, Annual Reports for Years 1995–2016, Table 10. https://ucr.fbi.gov/ucr-publications.
